# Human leukocyte antigen-G in solid tumors: from immunotolerance to immunotherapy

**DOI:** 10.3389/fimmu.2026.1696874

**Published:** 2026-04-10

**Authors:** Aifen Lin, Wei-Hua Yan

**Affiliations:** 1 Biological Resource Center, Taizhou Hospital of Zhejiang Province, WenZhou Medical University, Linhai, Zhejiang, China; 2 Key Laboratory of Minimally Invasive Techniques and Rapid Rehabilitation of Digestive System Tumor of Zhejiang Province, Taizhou Hospital of Zhejiang Province, WenZhou Medical University, Linhai, Zhejiang, China; 3 Key Laboratory of Human Leukocyte Antigen (HLA-G) Research and Antibody Development of Taizhou, Taizhou Hospital of Zhejiang Province, Wenzhou Medical University, Linhai, Zhejiang, China; 4 Medical Research Center, Taizhou Hospital of Zhejiang Province, WenZhou Medical University, Linhai, Zhejiang, China

**Keywords:** cancer, human leukocyte antigen-G, immune checkpoint, immunotherapy, isoform, receptor

## Abstract

Immune checkpoint-targeted immunotherapy has achieved unprecedented success, yet its limitations remain evident. Human leukocyte antigen-G (HLA-G), a novel immune checkpoint, exhibits restricted physiologic expression but is broadly expressed in various tumors, conferring systemic immune suppressive functions via different types of immune inhibitory receptors, and is associated with a poor prognosis for patients with cancer, making it an attractive tumor-site-agnostic candidate target for cancer immunotherapy. Since 2020, clinical trials employing different strategies of HLA-G-targeted immunotherapy for various advanced solid cancers have been conducted. Herein, the molecular characteristics of HLA-G, HLA-G-receptor binding interactions, and HLA-G-targeted preclinical investigations and clinical trials for solid cancer immunotherapy are highlighted, and the challenges associated with translating these findings into clinical settings are also discussed.

## Introduction

Since the first immune checkpoint inhibitor (ICI) against cytotoxic T lymphocyte-associated protein 4 (CTLA-4) was approved by the US Food and Drug Administration (FDA) in 2011, unprecedented success has been achieved in the field of immunotherapy for patients with cancer; however, limitations remain, such as the fact that only a subset of patients benefit clinically (1, 2). Inspired by these pioneering achievements, the exploration of novel ICIs to obtain better clinical benefits is increasing. Among these novel ICIs, human leukocyte antigen-G (HLA-G) is well acknowledged (3). HLA-G is a member of the non-classical HLA class I antigens (HLA-E, HLA-F, HLA-G, and HLA-H). Unlike the classical HLA class I antigens, which have been extensively investigated, the immunomodulatory roles and clinical significance of the non-classical HLA class I antigens in cancer biology, particularly HLA-G, have garnered increasing attention (4).

HLA-G was cloned by Geraghty et al. (5) in 1987, and its protein expression was first observed in cytotrophoblasts in 1990 (6). In the context of cancer, HLA-G expression in melanoma was first reported in 1998 (7). Over the past three decades, numerous studies have been conducted on HLA-G in cancer biology. Its pan-cancer expression and immunosuppressive function are closely associated with poor prognosis in patients with cancer, making HLA-G an ideal tumor-site-agnostic candidate target for solid cancer immunotherapy (8). In line with this, the first HLA-G-targeted clinical trial for advanced solid cancer immunotherapy was initiated in 2020. Briefly, HLA-G-targeted clinical trials employing various strategies for solid cancer immunotherapy have generated considerable enthusiasm (https://clinicaltrials.gov/search?cond=HLA-G).

In this review, updated molecular characteristics of HLA-G, HLA-G-receptor binding, and the potential of HLA-G-targeted immunotherapy are highlighted, and challenges in translating these findings into clinical settings are discussed.

## General features of HLA-G

**Figure 1:**
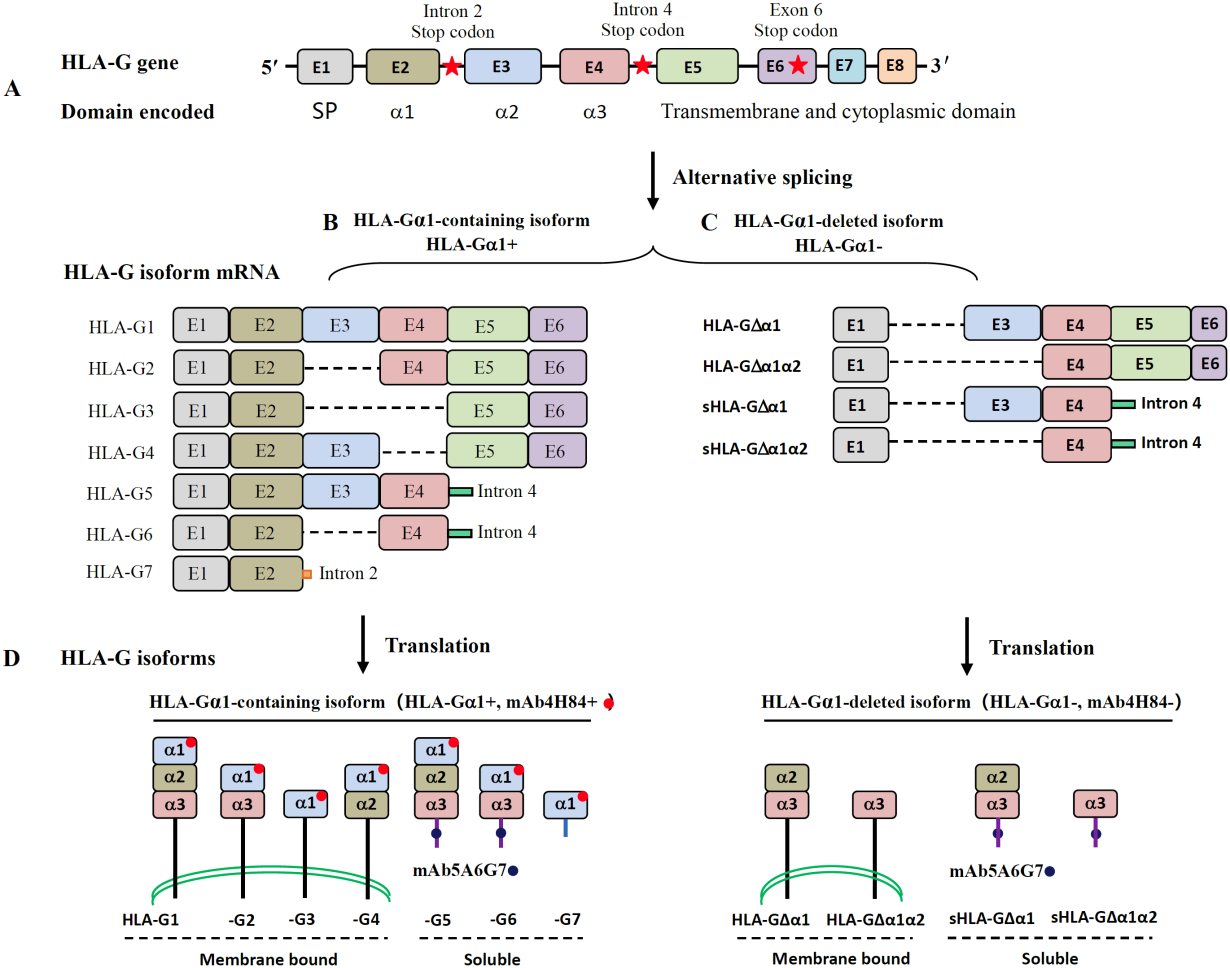
HLA-G isoforms generated by alternative splicing. **(A)** Overview of the HLA-G gene. E1~E8 represent the exons 1-8. E1 encodes the signal peptide (SP), and E2~E4 encode the α1, α2, and α3 domains, respectively. E5 encodes the transmembrane, and E6 encodes the cytoplasmic domain. **(B)** HLA-G α1-containing isoforms (HLA-Gα1+, including HLA-G1~HLA-G7). The membrane-bound isoforms HLA-G1, -G2, -G3, and -G4 are generated from an mRNA containing a stop codon (red star) in exon 6. The soluble isoforms HLA-G5 and HLA-G6 are generated from an mRNA with a stop codon in intron 4, and HLA-G7 generated a stop codon in intron 2. Pre-stop codons in intron 2 or 4 terminate transmembrane and cytoplasmic tail transcriptions. **(C)** HLA-G α1-deleted isoforms (HLA-Gα1-, including membrane-bound or soluble HLA-G△α1 and HLA-G△α1α2). The HLA-G△α1 and HLA-G△α1α2 are generated by the E2 and E2E3 spliced out mRNA due to a stop codon in exon 6, respectively. Soluble HLA-G△α1 and HLA-G△α1α2 are generated by the E2 and E2E3 spliced out mRNA and with a pre-stop codon in intron 4. **(D)** Schematic molecular structures of HLA-G isoforms. HLA-G α1-containing isoforms (HLA-Gα1+, including HLA-G1 -HLA-G7) can be probed with mAb4H84 (red dot), and the soluble HLA-G isoforms can be probed with mAb5A6G7 (blue dot).

HLA-G is a member of the non-classical HLA class I antigens (HLA-Ib, HLA-E, HLA-F, HLA-G, and HLA-H), which differ from classical HLA class I antigens (HLA-Ia, HLA-A, HLA-B, and HLA-C) in their limited genetic variation, restricted tissue distribution, and immunosuppressive functions. Although the intron-exon organization of HLA-G is similar to HLA-Ia and has a high degree of amino acid sequence similarity, HLA-G exhibits unique characteristics (**Figure 1**).

HLA-G has a stop codon in exon 6, and exons 7 and 8 are untranslated, resulting in a shorter cytoplasmic tail than that of HLA-Ia. Among the six exons in canonical HLA-G messenger RNA (mRNA), exons 1, 2, 3, 4, 5, and 6 encode the signal peptide, ectodomain α1, α2, and α3, transmembrane domain, and cytoplasmic tail, respectively (9). Multiple HLA-G isoforms with distinct structures are generated by alternative splicing, including α1-containing (HLA-Gα1+, e.g., HLA-G1–HLA-G7) and α1-deleted isoforms (HLA-Gα1-, e.g., HLA-G△α1 and HLA-G△α1α2) (10). Each α1-containing isoform (HLA-Gα1+) has a unique molecular structure, but all contain the ectodomain α1. Based on whether the transmembrane domain is retained, HLA-G includes four membrane-bound (HLA-G1, HLA-G2, HLA-G3, and HLA-G4) and three soluble isoforms (HLA-G5, HLA-G6, and HLA-G7) (11, 12). According to ectodomains α1, α2, and α3, HLA-G1 is the only full-length isoform with α1, α2, and α3 ectodomains. HLA-G2, HLA-G3, and HLA-G4 lack α1 and α2 owing to exon skipping; HLA-G2 contains α1 and α3; HLA-G3 contains only α1; and HLA-G4 contains α1 and α2 domains. Soluble isoforms HLA-G5 (containing α1, α2, and α3) and HLA-G6 (containing α1 and α3) are generated by mRNA with a stop codon in intron 4, which terminates the translation of both the transmembrane and cytoplasmic domains (13). HLA-G7 is a soluble isoform generated by mRNA with a stop codon in intron 2. Consequently, HLA-G7 is the shortest in amino acid length, containing only α1 followed by two amino acids encoded by intron 2 (14).

To date, two monoclonal antibodies (mAb 4H84 and mAb 5A6G7) for HLA-G detection are widely used. The mAb 4H84, generated with a peptide from 61–83 amino acids in the α1 domain, can detect all denatured heavy chains of HLA-G α1 domain-containing isoforms (HLA-Gα1+) (15). The mAb 5A6G7, generated with a peptide of 22 amino acids in the C-terminal sequence of HLA-G5 and HLA-G6, recognizes both native and denatured HLA-G5 and HLA-G6 (16). Although alternative pre-mRNA splicing is very common in eukaryotes (17), more unidentified HLA-G isoforms are expected. Novel HLA-G isoforms lacking α1 and α2, or other structural alternations, have been reported (12, 18).

## HLA-G receptors

**Figure 2:**
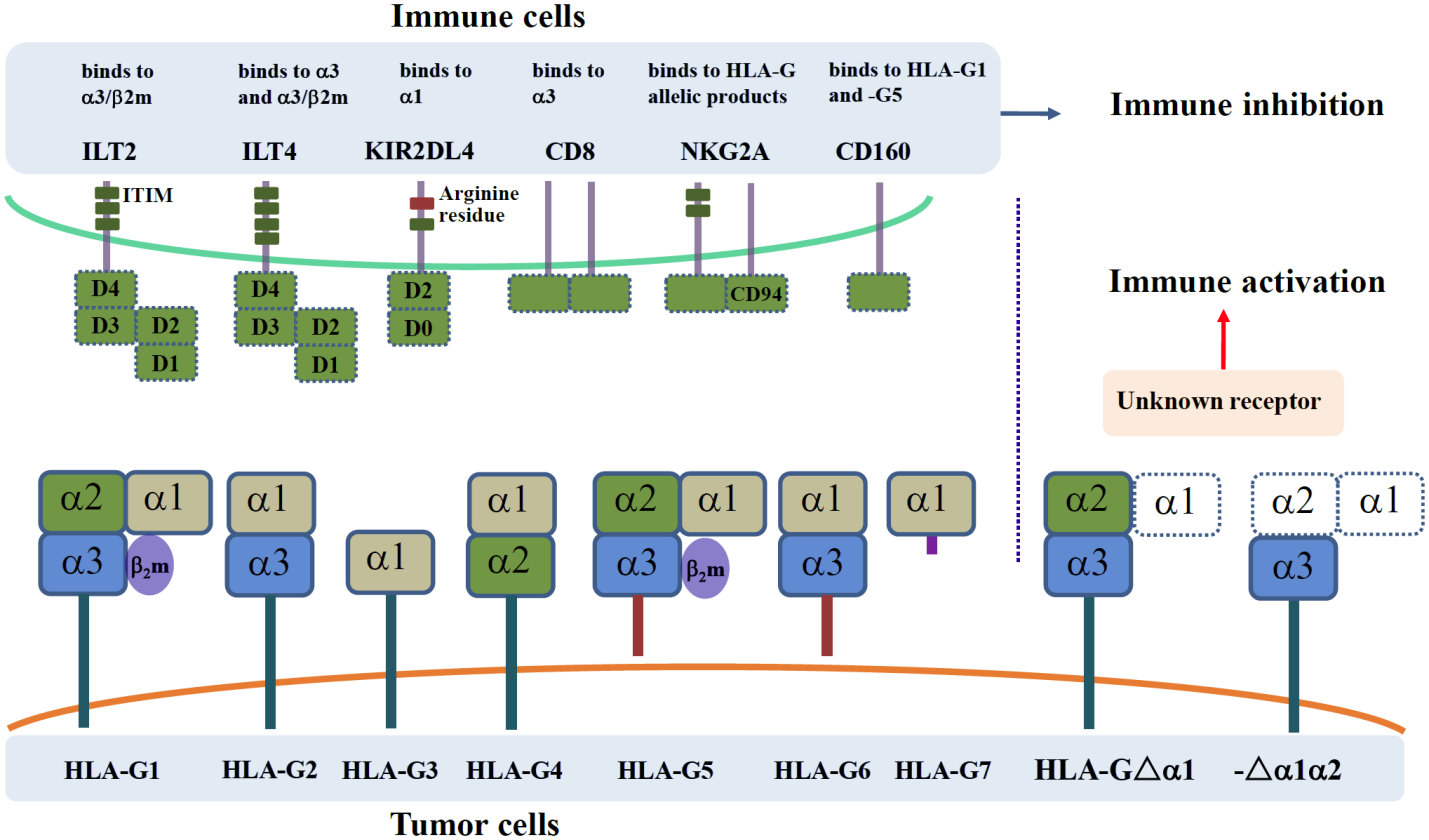
A schematic representation receptors for HLA-G receptors. Receptors ILT-2/LILRB1, ILT-4/LILRB2, KIR2DL4, CD8, NKG2A, and CD160 engage with corresponding domains of among HLA-G α1-containing isoforms (HLA-Gα1+). HLA-Gα1+ isoforms, including HLA-G1~HLA-G7, induce immune inhibitory signaling. HLA-G α1-deleted isoforms (HLA-Gα1-, including HLA-G△α1 and HLA-G△α1α2) induce immune stimulatory signaling while their receptor remains unknown yet.

Receptors for HLA-G recognition, including immunoglobulin-like transcripts 2 (ILT-2)/leukocyte immunoglobulin-like receptor B1 (LILRB1), ILT-4/LILRB2, and killer cell immunoglobulin-like receptor 2DL4 (KIR2DL4), were first reported in 1999 (19, 20). The roles of HLA-G expression in cancer through engagement with ILT-2**/**LILRB1, ILT-4/LILRB2, and KIR2DL4 have been investigated in numerous studies (21). Additionally, receptors CD8 and CD160 were reported to bind HLA-G, and natural killer gene 2A (NKG2A) could recognize specific HLA-G allele products (**Figure 2**) (22).

## Receptors ILT-2/LILRB1 and ILT-4/LILRB2

ILT-2**/**LILRB1 and ILT-4/LILRB2 contain four and three immunoreceptor tyrosine-based inhibitory motifs (ITIMs), respectively, in their cytoplasmic tails, which act in immune inhibitory signaling (23). ILT-2**/**LILRB1 and ILT-4/LILRB2 are differentially expressed in a wide range of immune cells, including T cells, B cells, natural killer (NK) cells, myeloid-derived suppressive cells (MDSCs), dendritic cells (DCs), and monocytes/macrophages (24).

ILT-2**/**LILRB1 and ILT-4/LILRB2 can recognize both HLA-Ia and HLA-Ib molecules, among which HLA-G binding has the strongest affinity. In addition, ILT-2**/**LILRB1 binding to HLA-G has a higher affinity than ILT-4/LILRB2 (25). HLA-G dimers have a structure that is more easily accessible for ILT-2**/**LILRB1 and ILT-4/LILRB2 binding, resulting in higher affinity for ILT-2**/**LILRB1 and ILT-4/LILRB2, and more efficient ILT-2**/**LILRB1 and ILT-4/LILRB2 inhibitory signaling than monomers (26, 27).

Structural analysis revealed that Ig-like ectodomains D1–D2 of ILT-2**/**LILRB1 and ILT-4/LILRB2 recognize both the α3 domain of HLA-G and the associated β2 microglobulin (β2m), while D3–D4 act as a scaffold for binding (28). Although ILT-2**/**LILRB1 and ILT-4/LILRB2 share high sequence identity (81%), their specificity for HLA-G binding is remarkably distinct. In addition, the binding sites differ between ILT-2**/**LILRB1 and ILT-4/LILRB2 in HLA-G recognition. ILT-2**/**LILRB1 residues Tyr^38^ and Tyr^76^ are tightly associated with the HLA-G residue Phe^195^, whereas ILT-4/LILRB2 residues Tyr^36^ and Arg^38^ bind the Phe^195^-Tyr^197^ loop in the α3 domain of HLA-G (29, 30). ILT-2**/**LILRB1 binding to HLA-G is β_2_m-dependent, as ILT-2**/**LILRB1 preferentially contacts β_2_m in the HLA-G/β2m complex, which can only bind β2m-associated isoforms (HLA-G1/β2m and HLA-G5/β2m). ILT-4/LILRB2 predominantly binds the α3 domain of HLA-G rather than the β2m, providing a β2m-independent binding mode that can bind either β2m-free or β2m-associated HLA-G isoforms, including β2m-free HLA-G1, HLA-G2, HLA-G5, HLA-G6, HLA-G1/β2m, and HLA-G5/β2m (31).

Functionally, both innate and adaptive immune responses can be inhibited by HLA-G/ILT-2 or HLA-G/ILT-4 engagement, including: (a) inhibition of immune cell proliferation, chemotaxis, and pro-inflammatory cytokine production (32, 33); (b) dampening cytotoxicity of T cells, tumor-infiltrating CD8^+^PD1^−^ILT-2^+^ T and invariant natural killer T (iNKT) cells, and cytotoxicity of NK cells (34–38); and (c) impairment of B-cell antibody production and antigen-presenting cell maturation and differentiation (39, 40). Furthermore, HLA-G1, ILT-2**/**LILRB1, and ILT-4/LILRB2 engagement contribute to regulatory T cell and MDSC expansion and polarize M1 macrophages toward M2 macrophages (41–43).

The immune suppression mediated by HLA-G, ILT-2**/**LILRB1, and ILT-4/LILRB2 interaction can be blocked by its antibodies or downregulation of HLA-G expression using mRNA interference and CRISPR/Cas9/HLA−G1 gene editing (44, 45). An anti-ILT-2 humanized monoclonal antibody, BND-22, specifically blocks the ILT-2 and HLA-G interaction, dramatically enhances antitumor immune responses, and improves the effects of anti-PD-1 or anti-epidermal growth factor receptor (EGFR) antibodies (46). ILT-4/LILRB2 blockade with the fully human monoclonal antibody MK-4830 was reported to enhance pro-inflammatory cytokines, including granulocyte-macrophage colony-stimulating factor (GM-CSF) and tumor necrosis factor α (TNFα) production, reduce tumor growth, and abrogate PD-1 resistance in patients with advanced solid tumors (NCT03564691) (47). These findings provide a strong rationale for HLA-G-targeted immunotherapy in patients with cancer.

## Receptor KIR2DL4

KIR2DL4 is an atypical receptor that exhibits both activating and inhibitory characteristics due to a unique positively charged arginine residue and an ITIM in its cytoplasmic tail (48). KIR2DL4 is mainly expressed on NK cells and is a specific receptor for HLA-G; residues Met (76) and Gln (79) in the HLA-Gα1 domain may be involved in KIR2DL4 recognition (19, 49).

The dichotomous functions of HLA-G and KIR2DL4 binding have been reported to depend on the NK cell status. In resting NK cells, HLA-G5 and KIR2DL4 interaction induces IFN-γ production without cytotoxicity via the DNA-PKcs endosomal signaling pathway. However, in activated NK cells, HLA-G1 and KIR2DL4 binding significantly inhibits NK cell cytotoxicity through SHP2 and phosphorylated tyrosine activation via a single cytoplasmic ITIM domain (50). Moreover, the presence or absence of HLA-G binding to KIR2DL4 was found to affect the functions of immune cells. KIR2DL4 expression on NK cells without HLA-G binding can promote the antitumor effects of activated NK cells in patients with colon cancer and induce apoptosis of HER2^+^ breast cancer cells (51, 52). In contrast, HLA-G binding to KIR2DL4 on mast cells facilitates HLA-G^+^ breast cancer metastasis (53). These findings echo the report by Zheng et al. (54), which showed that, without binding to HLA-G, KIR2DL4^+^NK cells could promote antibody-dependent cell-mediated cytotoxicity against HER2^+^ breast cancer cells, whereas HLA-G and KIR2DL4 signaling leads to resistance to trastuzumab treatment.

It is worth noting that the KIR2DL4 (CD158d) genotype influences expression and function in NK cells. Goodridge et al. (55) reported that two common KIR2DL4 alleles, with either 9 or 10 consecutive adenines (9A or 10A) in exon 6, could encode the transmembrane domain. The “9A” allele generates a secreted KIR2DL4 due to splicing out of the transmembrane region, which might result in a lack of cell surface KIR2DL4 expression. In contrast, the “10A” allele encodes a membrane-expressed receptor that is constitutively expressed on resting CD56^bright^ and CD56^dim^ NK cells. In these cases, KIR2DL4 sequencing might be needed to avoid functional cancellation when patients are treated with HLA-G-directed agents that block HLA-G/KIR2DL4 engagement and signaling.

## Other HLA-G receptors

HLA-G binding to CD8, CD160, and NKG2A receptors has also been reported in a few studies (56). The sHLA-G1 and CD8 interaction can induce apoptosis of CD8^+^ T cells and NKT cells via Fas/FasL signaling (57). CD160 is primarily expressed in cytotoxic CD8^+^ T cells, NK cells, mast cells, and activated endothelial cells (58). Binding of HLA-G1 to CD160 induces endothelial cell apoptosis and inhibits neoangiogenesis (59). NKG2A recognizes the HLA-G*01:04 encoded products (60). However, the functions and mechanisms underlying HLA-G and CD160 with HLA-G and NKG2A engagement remain poorly understood.

## HLA-G expression regulation and protein modification

**Figure 3:**
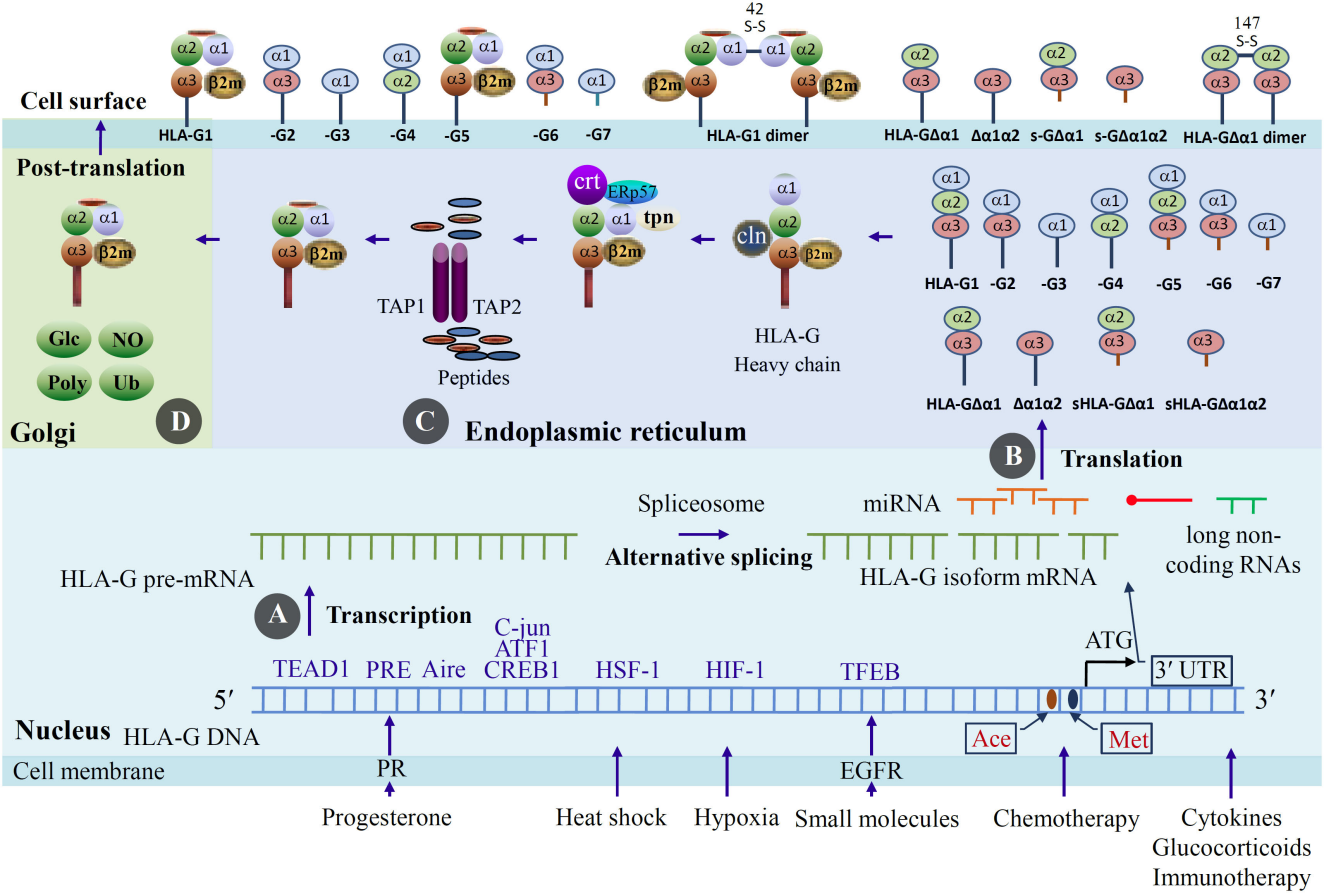
Regulation of HLA-G expression. **(A)***HLA-G* 5′UTR interacts with transcription factors activated by extracellular signals, such as hypoxia, heat shock, glucocorticoids, progesterone, cytokines, small molecules, and chemotherapeutic and immunotherapeutic agents. **(B)** miRNAs binding sites or polymorphisms in the *HLA-G* 3′ UTR modify mRNA stability or post-transcriptional regulation. Several long non-coding RNAs are reported to inhibit HLA-G specific miRNA binding. **(C)** The HLA-G/β_2_m/peptide conformer assembles in the endoplasmic reticulum through a stepwise process of folding, β_2_m assembly, and peptide loading, and **(D)** The HLA-G/β_2_m/peptide conformer translocated to the Golgi apparatus for protein modification and then transported to the cell surface. TEAD1, TEA domain family member 1; PR, progesterone receptor; PRE, progesterone response element; Aire, autoimmune regulator; ATF1, cyclic AMP-dependent transcription factor-1; C-jun, transcription factor encoded by the JUN gene; CRE, cAMP response element; CREB1, CAMP responsive element binding protein 1; HSE, heat shock element; HSF1, heat shock factor 1; EGFR, epidermal growth factor receptor; TFEB, transcription factor EB, PR, progesterone receptor; Ace, acetylation; Met, methylation; β2m, β2 microglobulin; Cln, calnexin; Crt, calreticulin; ERp57, endoplasmic reticulum p57; Tpn, tapasin; TAP, transporter associated with antigen presentation; Ub, ubiquitination; Glc, glycosylation; NO, nitration; Poly, polymerization.

The spatiotemporal expression of HLA-G is strictly regulated and fine-tuned under physiological conditions. To date, the distribution of HLA-G expression in normal tissues is very limited, including extravillous cytotrophoblasts, cornea, thymus, pancreatic islets, pituitary gland, testis, and prostate (61, 62). Multilevel mechanisms underlying the regulation of HLA-G expression have been documented (**Figure 3**). These include: (1) The 5’-untranslated regulatory region (5’-URR) exhibits features that are atypical compared to HLA-Ia, with many conserved regulatory boxes deleted, altered, or absent, including the interferon-stimulated response element, interferon-gamma-activated site, SXY module, and Enhancer A (63). Transcriptional regulation of HLA-G includes interactions between 5’-URR regulatory modules and various regulatory stimuli, such as hypoxia, progesterone, glucocorticoids, autoimmune regulator, cytokines, gene epigenetic modifications, and mRNA stability-related polymorphisms within the 3’ untranslated regions (3’-UTR) of HLA-G (64, 65). (2) Given that HLA-G transcripts are observed in various tissues, whereas protein distribution is very limited, highlights the importance of post-transcriptional regulation. HLA-G-specific miRNAs such as miR19a, miR-19b-1, miR-133a, miR-138-1-3p, miR-139-3p, miR-148a, miR-148b, miR-152, miR-548, miR-608, miR-628, miR744, miR-16-5p, miR-456-5p, miR-4488, miR-4753, miR-4516, miR-5096 (8, 63, 66–68), long non-coding RNAs VPS9D1-AS1, and HOX transcript antisense RNA (HOTAIR) have been reported to be involved in HLA-G expression (69, 70). (3) Small molecules and chemotherapeutic agents have been reported to enhance HLA-G expression, which affects treatment efficacy in patients with cancer. Induction of HLA-G expression in ovarian and breast cancer was observed following treatment with the poly (ADP-ribose) polymerase inhibitor (PARPi) niraparib, resulting in impaired cytotoxic activity of tumor-infiltrated NK cells. Wang et al. (71) reported that EGFR internalization is mediated by the PARPi niraparib, which activates AKT/mTOR signaling, enhances transcription factor EB transcriptional activity, and subsequently increases HLA-G expression. Furthermore, EGFR/NLRP3 inflammasome-activated-MAPK signaling is involved in the induction of HLA-G expression in oral cancer (72). The induction of cancer cell surface HLA-G expression by chemotherapeutic agents, such as pemetrexed, doxorubicin, temozolomide, gemcitabine, and carboplatin, has also been observed and may be mediated by the downregulation of DNMT1 and epigenetic regulation of the TAP-1 promoter (73, 74).

Notably, the nascent heavy chain of HLA-G is transported from the endoplasmic reticulum (ER) and Golgi apparatus to the cell surface in a strictly spatiotemporal stepwise order, including HLA-G heavy chain folding, light chain β_2_m assembly, and peptide loading, which requires the corresponding intracellular antigen-processing machinery components and chaperones in each step (75). The properly assembled HLA-G/β_2_m/peptide conformer is critical for HLA-G stability and cell-surface expression. The HLA-G α1–α2 ectodomains form the peptide-loading pocket, and a broad peptidome displayed by HLA-G has been reported (76, 77). Although the primary structures of HLA-G1 and HLA-G5 are predicted to conserve a peptide-binding groove and CD8-binding domain, antigen presentation to CD8+ T cells is unlikely to be the primary function of HLA-G (78). In line with this, Altadill et al. (77) revealed that HCMV-derived peptides loaded with HLA-G were irrelevant to TCR interactions, indicating that peptide binding might primarily contribute to the stability of HLA-G. Based on the molecular structure of HLA-G isoforms, only HLA-G1 and HLA-G5 contain α1–α2 domains and are associated with β_2_m, making it reasonable that HLA-G1 and HLA-G5 can be stably expressed. HLA-G1 and HLA-G5 expression have been investigated in many previous studies. However, data on the expression of membrane-bound HLA-G2, -G3, and HLA-G4, and soluble HLA-G6 and HLA-G7 remain controversial and require further investigation (79–81). Furthermore, post-translational protein modifications, such as monomer polymerization, phosphorylation, acetylation, ubiquitination, glycosylation, and proteolytic shedding of HLA-G1, have also been observed in previous reports, which may influence the interaction between HLA-G and receptor recognition and binding (82, 83), which may potentiallyreduce the affinity of anti-HLA-G antibodies (84).

## HLA-G expression in solid cancers

Innate immune cells are the first line of defense against tumors and are indispensable for the induction of adaptive T-cell-mediated antitumor responses. During tumor evolution, immunoediting exerts potent selection pressure against incipient cancers, leading to elimination or immune evasion through the acquisition of traits that disrupt antitumor immunity. The loss of HLA-Ia and HLA-II molecules is a frequent event in cancers to escape cytotoxic T cells and hamper responsiveness to ICI blockade (85, 86). In contrast, the aberrant induction of HLA-G during cancer immunoediting further inhibits both innate and adaptive antitumor immune responses, leading to a pro-neoplastic tumor microenvironment (TME) (87, 88).

Tumor-specific HLA-G expression and its clinical significance have been investigated in more than 30 types of histological malignancies since it was first identified in melanoma (11). Although significant inter- and intra-patient heterogeneity in HLA-G expression within and between tumors is commonly observed, its clinical significance has been demonstrated across a wide range of solid tumors, including bladder cancer, breast cancer, colorectal cancer (CRC), cervical cancer, endometrial carcinoma, esophageal cancer, Ewing sarcoma, gastric cancer, glioblastoma and glioma, hepatocellular cell carcinoma, lung cancer, lymphoma, Merkel cell carcinoma, nasopharyngeal carcinoma, oral squamous cell carcinoma, ovarian cancer, pancreatic adenocarcinoma, renal cell carcinoma (RCC), and thyroid carcinoma (8, 22, 89). Notably, Zhang et al. (90) recently reported for the first time that HLA-G expression in peritumoral fundic gland mucous neck cells, but not in tumor lesions, was associated with poor survival in patients with gastric cancer. HLA-G is heterogeneously present in different types of tumor lesions, and its expression in cancers is associated with malignant transformation, lower immune cell infiltration, distant metastasis, advanced disease stage, tumor recurrence, drug resistance, and poor prognosis (11). These findings were further solidified by a meta-analysis showing that HLA-G expression in solid tumors (n=4781) was significantly associated with poor overall survival (hazard ratio [HR] =2.09), particularly in patients with gastric (HR = 3.40), pancreatic (HR = 1.72), and colorectal cancers (HR = 1.55) (91). These findings differ from those for another non-classical HLA class I antigen, HLA-E. A study by Benitez Fuentes et al. (92) showed that an increased HLA-E expression was not significantly associated with overall survival in patients with solid cancers (HR = 0.913, n=911), whereas non-expression of HLA-E was significantly associated with improved disease-free survival (HR = 1.406, n=1068).

Moreover, the mechanisms underlying the promotion of cancer progression by HLA-G were also investigated. HLA-G1 expression significantly enhances the invasive potential and spheroid formation of ovarian cancer cells HO-8910 and OVCAR-3, resulting in distant organ metastasis and poor survival in Balb/c nu/nu mice (93, 94). Mechanistically, in addition to inducing NK cell cytolytic suppression, HLA-G1 specifically upregulates matrix metalloproteinase 15 (MMP-15), which contributes to the metastasis of HO-8910 ovarian cancer cells (93, 95). In an immunocompetent mouse model (42), HLA-G1 in the melanoma cell M8 was found to shift Th1/Th17 to Th2 cytokines and enhance MDSC proliferation, thereby providing an immunosuppressive microenvironment that permits rapid tumor growth before eventual rejection. With a mouse mammary 4T1/hHLA-G5^+^β2m^+^Balb/c mice model, results showed that HLA-G5 could impair hβ2m-elicited B-cell antibody production and enhance MDSC accumulation, thereby preventing hHLA-G5^+^β2m^+^ tumors from hβ2m-elicited immune rejection and allowing immunogenic 4T1/hHLA-G5^+^hβ2m^+^ tumors to grow (96). Moreover, HLA-G1 in ccRCC and melanoma tumor cells was found to modify key genes related to tumor development, angiogenesis, calcium flow, and mitochondria dynamics (97), indicating the multifaceted roles of HLA-G in tumor progression.

**Figure 4:**
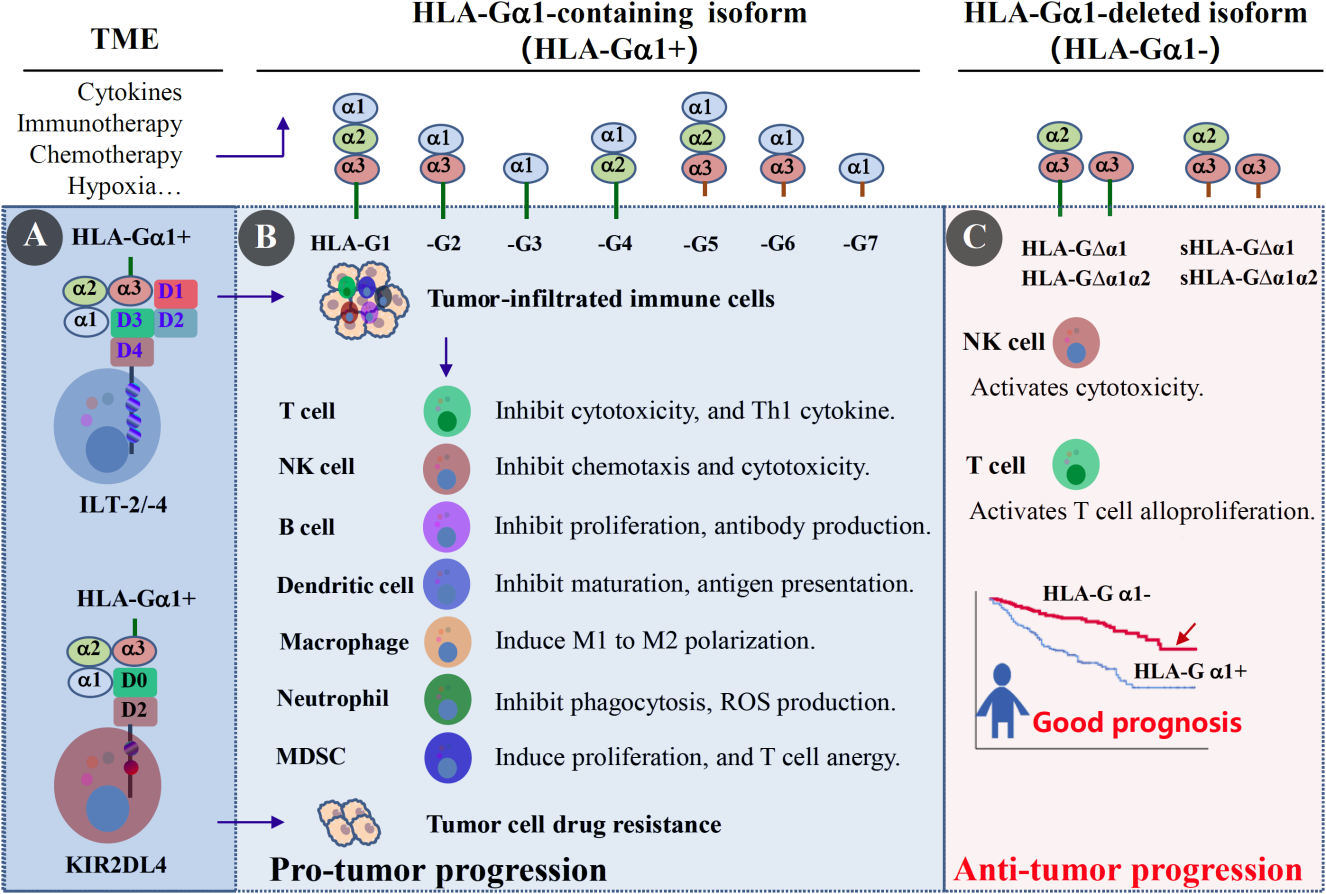
Immune modulation functions of HLA-G in cancer. **(A)** Receptor ILT-2/LILRB1 and/or ILT-4/LILRB2 express on tumor-infiltrated immune cells (T cells, NK cells, B cells, dendritic cells, macrophages, neutrophils, and MDSCs), and KIR2DL4 on NK cells in the tumor microenvironment (TME). Extracellular factors such as cytokines, chemotherapy, immunotherapy, and hypoxia can upregulate tumor expression of HLA-G α1-containing isoforms (HLA-G α1+, including HLA-G1~HLA-G7). **(B)** HLA-Gα1+/ILT-2/-4 mediated immune suppression in the TME, including (i) inhibiting immune cell proliferation, chemotaxis, and pro-inflammatory cytokine production; (ii) dampening T cell and NK cell cytotoxicity; (iii) impairing B cell antibody production and antigen-presenting cell maturation; and (iv) inducing Treg and MDSC expansion and M1 to M2 macrophage polarization. In addition, HLA-Gα1+/KIR2DL4 induces tumor cell drug resistance. **(C)** HLA-G α1-deleted isoforms (HLA-Gα1-, including membrane-bound or soluble HLA-G△α1 and HLA-G△α1α2) activate NK cell cytotoxicity and T cell alloproliferation and are associated with better prognosis. However, their receptors remain unknown.

Pre-mRNA alternative splicing is common in eukaryotes (17), and additional HLA-G isoforms are expected. Lin et al. (18) reported that HLA-G α1-containing (HLA-Gα1+) and HLA-G α1-deleted isoforms (HLA-Gα1-) were heterogeneously expressed in colorectal cancer lesions, with HLA-G α1-deleted isoforms (HLA-Gα1-) being associated with a better prognosis than HLA-G α1-containing isoforms (HLA-Gα1+). This observation was further supported by the finding that, in contrast to the immune inhibitory function of α1-containing isoforms (HLA-Gα1+), Tronik Le Roux et al. (98) recently reported that HLA-G△α1 has immunostimulatory function. Thus, further studies are needed to explore the clinical significance of co-expression of HLA-G α1-containing (HLA-Gα1+) and HLA-G α1-deleted isoforms (HLA-Gα1-) in cancers to optimize potential strategies for HLA-G-targeted immunotherapy (**Figure 4**).

## HLA-G-targeted immunotherapy for solid cancers

**Figure 5:**
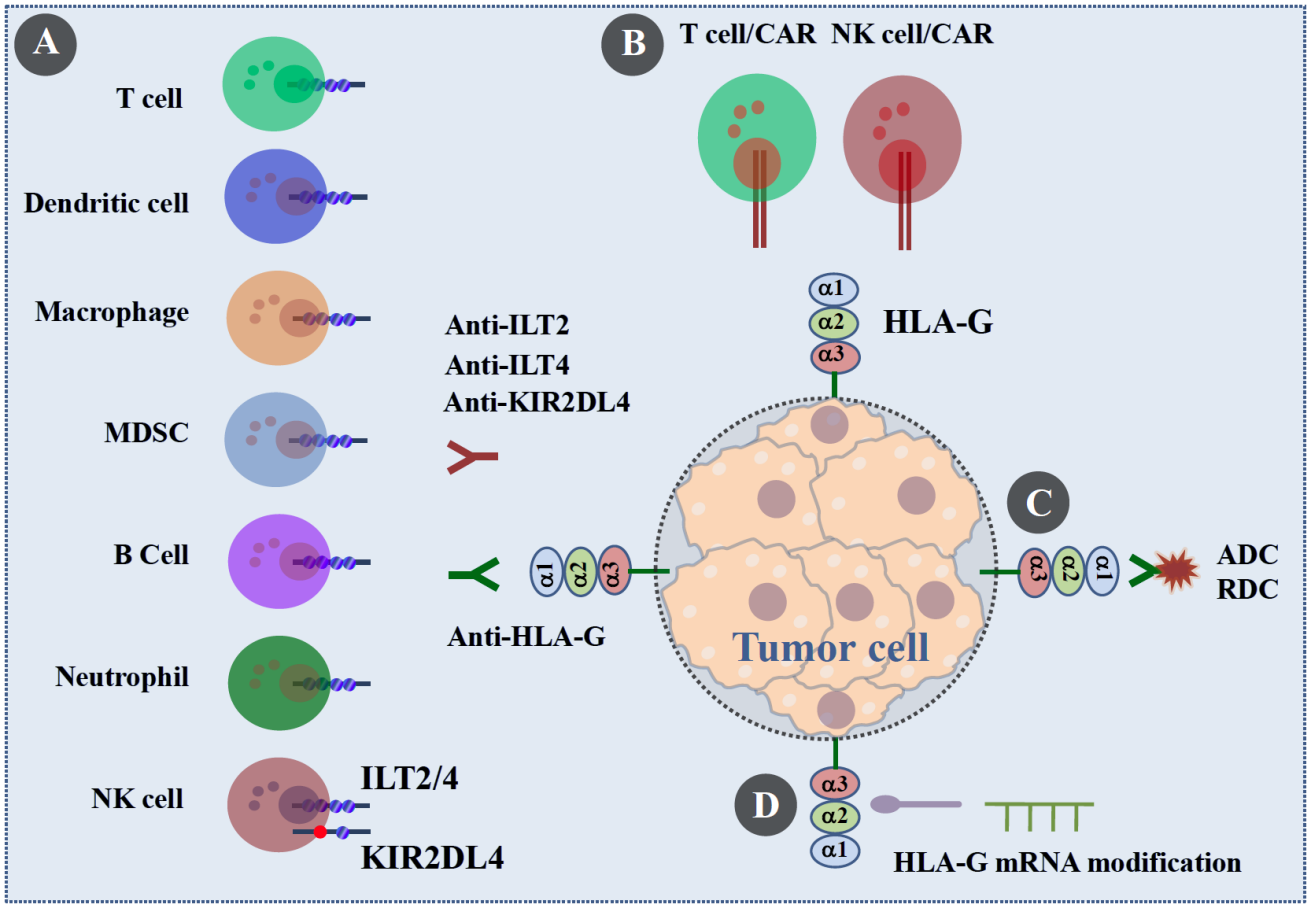
Perspectives on HLA-Gα1+ isoform (including HLA-G1~HLA-G7) targeted immunotherapy in solid cancers. HLA-Gα1+-targeted cancer immunotherapy include: **(A)** monoclonal antibodies/inhibitors targeting HLA-Gα1+/ILT-2/-4 and HLA-Gα1+/KIR2DL4 signaling; **(B)** HLA-G mAb-derived CAR-T and CAR-NK targeting HLA-Gα1+ to prevent tumor growth and metastasis and prolong survival; **(C)** HLA-G antibody-drug conjugates (ADCs) or radionuclide drug conjugates (RDC) delivered to HLA-Gα1+ positive tumor cells; and **(D)** downregulate HLA-Gα1+ and upregulate HLA-Gα1- isoform mRNA to enhance antitumor immune responses.

Given the plethora of evidence accumulated over the past three decades, HLA-G has been well established as a novel ICI and an attractive neoantigen and tumor-site-agnostic candidate target. Different potential strategies for HLA-G-targeted preclinical studies and clinical trials in advanced solid cancer immunotherapy are currently being evaluated or are anticipated (**Figure 5**) (8, 99).

## Preclinical studies of HLA-G-targeted solid cancer immunotherapy

Anna et al. (100) established two anti-HLA-G/CAR-T using the single-chain variable fragment (scFv) derived from anti-HLA-G mAbs LFTT1 and 15E7: anti- HLA-G/CAR-T/LFTT1 (specific for β2m-associated HLA-G1/-G5) and anti-HLA-G/CAR-T/15E7 (specific for β2m-free HLA-G1, -G2,-G5, and-G6). Using K562-G1 (expressing both β2m-free and β2m-associated HLA-G1) and JEG-3 (expressing β2m-associated HLA-G1) as target cells, both CAR-LFTT1 and CAR-15E7 specifically targeted K562-G1 and JEG-3 cells. Notably, both CAR-LFTT1 and CAR-15E7 differentiated into memory effectors and exerted lasting functions against, or eradicated, HLA-G-positive tumors in the NOD/SCID mouse model.

Another anti-HLA-G/CAR-T (specific for HLA-G1, HLA-G2, HLA-G4, HLA-G5, and HLA-G6) was effective against oral squamous carcinoma co-expressing HLA-G/wild-type (EGFR^WT^) or mutated variants (EGFR^19del^, EGFR^L858R^, and EGFRvIII) *in vitro* and reduced HLA-G^+^EGFR^+^ tumor growth and metastasis in NOD/SCID mice *in vivo* (72).

Using an HLA‐G nanobody linked to a bispecific PD‐L1/CD3ϵ T‐cell engager (BiTE), Huang et al. (101) revealed that Nb‐CAR.BiTE/CAR-γδT could effectively target PD-L1 or HLA-G/PD-L1 co-expressing solid tumor cells *in vitro* and eliminate tumors while prolonging survival in HLA‐G^high^/PD‐L1^high^MDA‐MB‐231 or PD‐L1^high^A549 cell xenograft PBMC‐humanized NSG mouse models (PBMC‐CDX‐NSG). Additionally, the same study group recently reported that a trispecific nanobody construct (Nb-TriTE, specific for HLA-G, PD-L1, and CD3ε) could also activate the cytotoxic capability of macrophages, T cells, and PBMCs against PD-L1 or HLA-G expressing A549, MDA‐MB‐231, U‐87 MG, SK‐OV‐3, and FaDu cells. Furthermore, Nb-TriTE could redirect T cells to tumor sites and render potent antitumor effects in the A549 tumor cell‐derived PBMC‐CDX‐NSG model (102).

Jan et al. (73) demonstrated that anti-HLA-G/CAR-NK cells (with unspecified specificity) could specifically target a panel of HLA-G-positive tumor cells by decreasing phosphor-SHP-1 while upregulating the phosphor-Syk/Zap70 signaling pathway, thereby triggering NK cell killing activity both *in vitro* and *in vivo*.

## Clinical trials for HLA-G targeted solid cancer immunotherapy

The aforementioned preclinical studies provide strong evidence for future clinical trials based on HLA-G alone and HLA-G combined with other checkpoints for solid cancer immunotherapy (https://clinicaltrials.gov/search?cond=HLA-G; **Table 1**).

**Table 1:** Current HLA-G related clinical trials.

NCT	Title	Drug	Drug target	Lesion HLA-G assay mAb (clone)	HLA-G positivity cut-off and location	β2m dependence	Targeting HLA-G isoform assumption	Summary
NCT04485013	TTX-080 HLA-G antagonist in subjects with advanced cancers	TTX-080 (anti-HLA-G mAb)	A fully humanized HLA-G mAb blocking the HLA-G, ILT-2 and ILT-4 interaction.	NR	NR Location: primary or metastasis.	NR	HLA-G1, HLA-G2, HLA-G5 and HLA-G6	
NCT04991740	A study of JNJ-78306358 in participants with advanced stage solid tumors	(HLA-G x CD3 BiTE)	A bispecific antibody binding to CD3 on T cells and HLA-G on cancer cells. JNJ-78306358 binds an epitope on α3 domain of HLA-G and competes with ILT-2/-4.[reference 105]	Tumor HLA-G (mAb 4H84)	HLA-G positivity (H-score > 0). [reference 104] Location: primary.	NR	HLA-G1, HLA-G2, HLA-G5 and HLA-G6	
NCT06380816	A phase I/​II trial of UCB4594 in participants with advanced cancer	UCB4594 (anti-HLA-G mAb)	NR	NR	NR Location: primary.	NR	N/A	
NCT04300088	A prospective study of the relevance of the HLA-G immune checkpoint in cancer immunotherapy (GEIA)	anti-PD(L)1 with or without anti-CTLA4	N/A	Tumor HLA-G (IHC, NR), sHLA-G (ELISA)	NR Location: primary.	NR	N/A	
NCT05672459	A safety and efficacy study of HLA-G-targeted CAR-T cells IVS-3001 in subjects with previously treated advanced HLA-G-positive solid tumors	IVS-3001 autologous CAR-T targeting HLA-G	An autologous anti-HLA-G CAR-T cells targeting HLA-G positive solid tumors.	Tumor HLA-G (mAb 4H84)	NR Location: primary.	NR	N/A	
NCT05769959	Study of RO7515629 in participants with HLA-G positive solid tumors	RO7515629 (anti-HLA-G mAb)	NR	NR	NR Location: primary or metastasis.	NR	N/A	
NCT	Title	Phase	Patient selection	Study type	Study design	N	Status	Brief summary
NCT04485013	TTX-080 HLA-G antagonist in subjects with advanced cancers	1a/1b	Adult patients with advanced refractory/resistant solid malignancies, including metastatic colorectal cancer (mCRC) patients	Intervention	Open label, non-randomized, parallel assignment	240	recruiting	To assess safety, tolerability of TTX-080 monotherapy, combination with either Pembrolizumab, Cetuximab or FOLFIRI plus Cetuximab
NCT04991740	A study of JNJ-78306358 in participants with advanced stage solid tumors	1	Adult patients with advanced solid Tumors	Intervention	Open label, non-randomized, sequential assignment	39	Completed	To assess safety, pharmacokinetics, biomarkers, immunogenicity, and efficacy of JNJ-78306358.
NCT06380816	A phase I/​II trial of UCB4594 in participants with advanced cancer	1/2	Adult patients with advanced solid cancers	Intervention	Open label, non-randomized, sequential assignment	167	recruiting	To assess safety, tolerability, pharmacokinetics and anti-tumor activity of UCB4594 alone and in combination with anti-cancer treatments.
NCT04300088	A prospective study of the relevance of the HLA-G immune checkpoint in cancer immunotherapy (GEIA)	–	Adult patients with advanced solid cancer treated with anti-PD(L)1 immunotherapy with or without anti-CTLA4 immunotherapy.	Observation	Prospective cohort	281	Not yet recruiting	To assess the impact of HLA-G expression on the efficacy of immunotherapy (anti-PD-1/PD-L1 with or without anti-CTLA4).
NCT05672459	A safety and efficacy study of HLA-G-targeted CAR-T cells IVS-3001 in subjects with previously treated advanced HLA-G-positive solid tumors	1/2a	Adult patients with locally advanced unresectable or metastatic HLA-G+ select solid tumor malignancy.	Intervention	Open label, non-randomized, Single Group Assignment	117	Recruiting	To assess safety, tolerability, pharmacokinetics and clinical activity of the IVS-3001.
NCT05769959	Study of RO7515629 in participants with HLA-G positive solid tumors	1	Adult patients with unresectable and/or metastatic HLA-G positive solid tumors.	Intervention	Open label, sequential assignment	3	Terminated	To assess safety, tolerability, pharmacokinetics, immune response and preliminary anti-tumor activity of RO7515629 monotherapy.

NR, not reported; N/A, not applicable.

HLA-G-targeted solid cancer immunotherapy has attracted great enthusiasm since the first clinical trial (NCT04485013) was launched in 2020. This phase I clinical trial evaluated the safety and efficacy of the HLA-G antagonist TTX-080 monotherapy (a fully humanized HLA-G mAb blocking the HLA-G, ILT-2**/**LILRB1, and ILT-4/LILRB2 interaction) and included a planned phase Ib trial to test the efficacy of combining TTX-080 with the PD-1 inhibitor pembrolizumab/EGFR inhibitor cetuximab in patients with advanced refractory/resistant solid malignancies, including head and neck squamous cell carcinoma, colorectal cancer, and triple-negative breast cancer. Clinical trial NCT05769959 is evaluating the anti-HLA-G antibody RO7515629, and NCT06380816 was designed to evaluate the anti-HLA-G antibody UCB4594 in patients with various advanced or metastatic HLA-G-positive solid tumors. However, the clinical trial was terminated by the sponsor on March 19, 2024. In a phase I/II trial (NCT05672459) evaluating anti-HLA-G/CAR-T cells (IVS-3001), safety and clinical activity are currently being evaluated at dose level 3 in patients with advanced or metastatic HLA-G-positive (detected by mAb4H84) solid tumors (103).

The clinical trial (NCT04991740) evaluating a bispecific antibody, JNJ-78306358, which binds to CD3 and α3 domain-containing HLA-G isoforms (detected by mAb4H84) was completed on February 9, 2023, and the results have been reported (104, 105). The HLA-G/CD3 bsAb JNJ-78306358 contains a high-affinity HLA-G binding anti-HLA-G scFv and a lower-affinity CD3ϵ binding Fab domain. In this clinical trial, 12 of 25 patients were HLA-G-positive. Although no objective responses were observed [per Response Evaluation Criteria in Solid Tumors (RECIST) version 1.1], half of the treated patients (17/34) showed disease stabilization, and two patients maintained stable disease for > 40 weeks. Unfortunately, the trial was terminated due to disease progression, which might have resulted, as described by the authors, from immune-associated toxicities that prevented dose escalation of JNJ-78306358 to reach efficacious levels, in addition to a high frequency of anti-drug antibody (ADA) development, which reduced drug exposure and had significant neutralization potential. The significance of HLA-G expression in determining the efficacy of JNJ-78306358 was not addressed in the study, leaving unresolved whether expression of α3 domain-containing HLA-G isoforms is associated with the efficacy of JNJ-78306358 treatment (104).

Although detailed data are still limited, clinical trials in different settings are expected to provide insights into the future clinical application of HLA-G antibody monotherapy or combination therapies with other ICIs, potentially leading to improved clinical outcomes for patients with cancer.

## HLA-G antibody-drug conjugates

Apart from conventional treatments with mAbs that directly block ICI signaling, mAbs can also serve as antibody-drug conjugates (ADCs), an antitumor nanoplatform that can precisely guide “homing missiles” for cancer therapy (106). Zhang et al. (107) showed that the HLA-G mAb MEM-G09 (specific for HLA-G1 and HLA-G5) could precisely guide the methotrexate (MTX)-loaded nanobubbles (mAbHLA-G/MTX/PLGA NBs) to the HLA-G1-positive choriocarcinoma cell line JEG-3. The authors reported that mAb HLA-G/MTX/PLGA could directly target HLA-G-positive JEG-3 cells *in vitro* and JEG-3-derived choriocarcinoma mice model *in vivo*, and that mAb MEM-G09/HLA-G binding could efficiently and precisely guide loaded MTX to eradicate HLA-G-positive residual cancer cells.

As HLA-G is a pan-cancer-specific immune checkpoint, chemotherapeutic drugs, radionuclide drugs, and even spliceosome modulators and RNA polymerase inhibitor payloads on ADCs can be directly delivered to HLA-G-positive tumor cells with more efficacy, specificity, and less toxicity. Moreover, such strategies may shift the predominance of immune inhibition to stimulate HLA-G isoforms, making HLA-G-targeted ADC a new pan-cancer-specific alternative modality for cancer therapy.

## Challenges of HLA-G-targeted immunotherapy for solid cancers

Novel strategies for cancer immunotherapy that block the pan-cancer-specific HLA-G, ILT-2**/**LILRB1, and ILT-4/LILRB2 signaling axis have generated significant enthusiasm since 2020. Nevertheless, several challenges must be addressed to achieve precise HLA-G-targeted cancer immunotherapy.

First, whether HLA-G isoforms other than HLA-G1 and HLA-G5 are mature, functional on the cell surface, and secreted into the tumor microenvironment remains to be further investigated (79, 81). In addition, the immune-inhibitory HLA-G α1-containing (HLA-Gα1+, including HLA-G1~HLA-G7) and immune-stimulatory HLA-G α1-deleted isoforms (HLA-Gα1-, including HLA-G△α1 and HLA-G△α1α2) are highly heterogeneously co-expressed in cancers (18, 98). Currently, HLA-G therapeutic antibodies/inhibitors are generated against epitopes within the α1–α3 domains of HLA-G (HLA-G1, HLA-G2, HLA-G5, and HLA-G6) to block HLA-G, ILT-2**/**LILRB1, and ILT-4/LILRB2 signaling. Although α3 domain-containing HLA-G α1-deleted isoforms (HLA-Gα1-) are independent of ILT-2**/**LILRB1, HLA-G antibodies/inhibitors targeting α3 domains may also block the immune-stimulatory functions of HLA-G α1-deleted isoforms (HLA-Gα1-), potentially impairing the efficacy of HLA-G-targeted cancer immunotherapy. Moreover, mAb 4H84 recognizes only one domain of HLA-G, which cannot avoid false-negative staining for the HLA-G α1domain-deleted isoforms (HLA-Gα1-) (12, 18). In this context, with the successful generation of mAbs for HLA-G2/6 and HLA-G1/4/5 isoforms, Zhang et al. (108) first demonstrated that HLA-G2/6, but not HLA-G1/4/5, expression is an independent prognostic indicator of poor survival in patients with CRC. Therefore, the development of isoform-specific antibodies is critical for establishing the relevance of individual isoforms within the HLA-G variant family in health and disease.

Second, HLA-G may upregulate other immune checkpoints in cancers or increase ILT-2**/**LILRB1 and ILT-4/LILRB2 expression in immune cells, thereby leading to a more profoundly immunosuppressive tumor microenvironment (3). The sHLA-G1 and ILT-2**/**LILRB1 binding has been reported to increase PD-1, CTLA-4, TIM-3, and CD95 expression exclusively in ILT-2^+^CD8^+^ T cells (36). Functionally, the HLA-G and ILT-2**/**LILRB1 interaction specifically inhibits CD8^+^ILT-2^+^ T cells or CD4^+^ILT-2^+^ (Tbet^+^Perforin^+^KLRG1^+^NKp80^+^GPR56^+^) cytotoxic T cells, but not peripheral CD8^+^ILT-2^-^ T cells or CD8^+^PD-1^+^tumor infiltrated T cells (34, 37). These findings suggest that sHLA-G induces a pronounced immunosuppressive phenotype in immune cells, counteracting other immune checkpoint-targeted immunotherapies. To address this, a prospective clinical trial (NCT04300088) is evaluating whether tumor HLA-G and peripheral sHLA-G expression affect clinical responses to anti-PD-1, PD-L1, and anti-CTLA-4 therapies. This study demonstrates the direct clinical relevance of HLA-G expression in patients undergoing immune checkpoint-targeted immunotherapy, thus optimizing the design of precise therapeutic protocols in terms of HLA-G to obtain better clinical benefits.

## Conclusions

Significant breakthroughs have been achieved since the development of the first ICI of CTLA-4 for advanced solid cancer immunotherapy; however, only a subset of patients have clinically benefited from it. To overcome these limitations and achieve better therapeutic outcomes, new therapeutic agents with novel targets are required. HLA-G, characterized by restricted physiological expression, broad presence across many tumors, immunosuppressive properties, and strong association with poor prognosis of patients with cancer, represents a promising target for cancer immunotherapy. However, challenges must be addressed to enable precise HLA-G-targeted immunotherapy, including: (1) clarifying whether HLA-G isoforms beyond HLA-G1 and HLA-G5 are matured, transported to cell surface, or secreted into TME with biological functionality; (2) given multiple and heterogeneous HLA-G isoforms could be expressed in cancers, HLA-G isoform-specific antibodies are critically necessary to quantify or identify their cellular localization, to define the profiles of HLA-G isoforms expression, and to evaluate relevance of HLA-G isoforms in cancers; (3) systematically evaluating potential side effects of HLA-G-targeted therapies on HLA-G positive normal tissues, such as immune-privileged tissues; and (4) elucidating the mechanisms and clinical significance of crosstalk between among HLA-G and other ICIs within the TME. Despite these challenges and the current lack of robust clinical evidence, HLA-G could be a promising candidate target for solid cancer immunotherapy.
